# Work pressure, coping styles and occupational burnout among Chinese police officers: a meta-analytic review

**DOI:** 10.1186/s40359-024-01779-6

**Published:** 2024-05-16

**Authors:** Senlin Zhou, Miaomiao Li, Siru Chen, Daokui Jiang, Ying Qu, Xizheng Xu

**Affiliations:** 1 Hunan Police Academy, Changsha, 410138 China; 2 School of Economics and Management, Shanghai University of Political Science and Law, Shanghai, China; 3 School of Business, Hunan Institute of Technology, Hengyang, China; 4 Business School, Shandong Normal University, Jinan, China; 5 Department of Psychology, Liaoning Normal University, Dalian, China

**Keywords:** Work pressure, Occupational burnout, Coping styles, Meta-analytic, Meta-analytic structural equation modeling (MASEM)

## Abstract

The present study conducted a comprehensive meta-analysis to systematically review the relationship between occupational burnout and work pressure among Chinese police officers. Additionally, the study explored the mediating role of coping styles using a meta-analytic structural equation model. The investigation involved a thorough search of CNKI, PubMed, PsychInfo, Web of Science, and Google Scholar databases, resulting in the identification of a total of 39 studies with 124 effect sizes and 14,089 police officers. The findings revealed a positive correlation between work pressure and occupational burnout among Chinese police officers (*r* = 0.410, 95% CI = [0.347, 0.469]). Furthermore, negative coping styles mediate the relationship between work pressure and occupational burnout. Importantly, these conclusions held true across various work regions for police officers. These results provide insights into the relationship magnitude between work pressure and occupational burnout in Chinese police work and shed light on the underlying mechanisms. Based on these findings, it is recommended that interventions focusing on reducing work pressure and fostering positive coping styles be implemented to mitigate occupational burnout among police officers.

**Supplementary Information:**

The online version contains supplementary material available at 10.1186/s40359-024-01779-6.

## Introduction

Occupational burnout has emerged as a critical concern across various professions, especially those characterized by high-stress environments [1]. The law enforcement field is acutely susceptible to burnout, given the inherently demanding nature of police work compounded by substantial societal expectations. Officers face the dual challenges of maintaining public safety while safeguarding their mental well-being amidst inherent job hazards, the need for constant vigilance, and the weight of public accountability [2]. This phenomenon is particularly pronounced among Chinese police officers due to unique cultural, social, and operational contexts that amplify the pressures they encounter [3]. The rigorous demands placed on them, combined with distinct societal norms and the comprehensive scope of their duties, significantly heighten their burnout risk. This not only threatens officers’ health and well-being but also impacts their job performance and public safety. Consequently, the prevalence of burnout among Chinese police underscores an urgent need to investigate this issue and identify effective mitigation strategies to enhance law enforcement efficiency and public service delivery.

While a growing body of literature examines work stress and burnout among global police forces, the findings present inconsistencies [4]. Some studies indicate a strong positive correlation between work pressure and burnout [5], while others report weaker or non-significant relationships [6]. This discrepancy suggests complex interplays among various factors influencing burnout, which remain underexplored, particularly within the unique Chinese policing context. Most existing research has been conducted in Western settings [7, 8], leaving a gap in understanding how China’s distinct cultural and systemic differences might shape the experiences and determinants of police burnout.

This study aims to systematically investigate the intricate relationships between work pressure, coping strategies, and occupational burnout specifically among Chinese police officers. Recognizing the pressing need to examine these dynamics against China’s unique cultural and occupational backdrop, the key objectives are: (a) To quantify the strength and direction of the relationship between work pressure and burnout. (b) To identify potential moderating variables that may influence this relationship. (c) To explore the mediating role of coping strategies in this relationship.

### Literature review

#### Theoretical basis

The Job Demands-Resources (JD-R) model [9] offers a robust explanatory framework for the link between work stress and job burnout. This model suggests that the imbalance between various job demands (such as high workload, emotional demands, and working hours) and available job resources (like social support, and autonomy) during work processes is a key precursor to job burnout. When job demands consistently exceed the coping resources available to an individual, it inevitably leads to excessive depletion and energy drain, ultimately triggering symptoms of job burnout such as emotional exhaustion. The JD-R model elucidates how work stress, as a form of job demand, affects burnout by depleting an individual’s psychological and physiological resources [10].

Conservation of Resources (COR) theory [11] further supplements the critical role of resources in coping with stress. This theory posits that stress arises from the threat of resource loss or the inability to obtain expected returns on resources. When individuals invest significant resources (such as time, and energy) to cope with work stress but the returns are insufficient to compensate for the losses, long-term net loss of resources leads to stress and burnout. In the context of law enforcement work, police officers often face the dilemma of resource scarcity, and lack sufficient personal resilience, social support, professional training, and organizational support [12, 13]. This scarcity of resources exacerbates their risk of job burnout.

Lastly, Stress and Coping Transactional Theory [14] provides a perspective for understanding the role individuals play in the stress process. This theory emphasizes that the level of stress and the choice of coping strategies depend on an individual’s subjective assessment of specific environmental demands. When police officers assess job demands as exceeding their coping abilities, they experience higher stress and may adopt inappropriate coping mechanisms (such as cutting corners or venting emotions), thereby exacerbating job burnout. Conversely, if they realistically assess their resources and adopt appropriate proactive coping strategies (such as seeking support or problem-solving), it may reduce the impact of stress on job burnout.

By integrating these three major theories, this study aims to comprehensively clarify the network of relationships and complex mechanisms between work stress, coping strategies, and job burnout.

#### Experimental studies

Research on occupational burnout, especially within high-stress professions like law enforcement, has yielded a diverse array of findings that often lack consistency [6]. This divergence across studies highlights gaps in our collective understanding of burnout’s causes, progression, and consequences. The inconsistencies are particularly evident when exploring the nexus between work-related stress and burnout, where factors like work pressure, coping strategies, and organizational support have been variously linked to burnout outcomes [15, 16].

Globally, studies have taken diverse approaches to examining stress and burnout [17, 18], offering valuable perspectives on their prevalence, contributing factors, and impact on law enforcement. While Western research often emphasizes job demands and lack of resources as principal burnout drivers [6], Asian studies tend to highlight additional individual differences and societal influences [19, 20]. This variance points not only to burnout’s complexity but also to how individual and societal factors may modulate these relationships. The role of coping strategies mediating stress and burnout has also been extensively investigated, with findings suggesting both adaptive and maladaptive mechanisms significantly influence burnout development [21, 22]. However, the extent to which different strategies mediate this relationship warrants further exploration across cultures. Additionally, factors like gender and work reagin may moderate the stress-burnout relationship, though findings on their impacts have been inconsistent [23, 24]. This inconsistency necessitates a nuanced understanding considering the interplay of multiple factors.

Given these complexities, a comprehensive meta-analysis is needed to synthesize existing research and offer clearer insights into burnout mechanisms, particularly in law enforcement contexts. By integrating data across studies, meta-analyses can elucidate burnout prevalence, work stress impacts, coping strategy mediation effects, and organizational/personal moderating factors among police officers. This approach addresses literature gaps while laying the groundwork for mitigating burnout risks through targeted interventions tailored to law enforcement agencies’ unique cultural and operational contexts.

### Current study

The current study aims to comprehensively clarify the network of relationships and complex mechanisms between work stress, coping strategies, and job burnout.

The specific objectives of the current study are as follows: (a) Quantifying the actual effect size between stress and job burnout; (b) Exploring the mechanism of this relationship — The mediating role of coping strategies; (c)Investigating moderating variables that may influence the relationship.

Based on the objectives outlined in the current study, we propose the following three hypotheses to guide our investigation into the relationships and mechanisms between work stress, coping strategies, and job burnout:

#### Hypothesis 1

The work pressure experienced by policemen is significantly and positively associated with occupational burnout.

#### Hypothesis 2a

The influence of police work pressure on occupational burnout is mediated by positive coping styles.

#### Hypothesis 2b

The influence of police work pressure on occupational burnout is mediated by negative coping styles.

It is important to note that due to the absence of adequate theoretical backing for the moderating variables that might affect the connection between work stress and job burnout, we will refrain from establishing specific hypotheses for these moderating variables. Instead, the analysis of these variables will be conducted with an exploratory approach.

## Method

Following the guidelines of the Preferred Reporting Items for Systematic Reviews and Meta-Analyses (PRISMA) 2020 statement [25], the search for relevant articles was conducted using a multi-step approach. The initial searches were performed in January 2023 and covered publications from database inception to December 2022. Firstly, two authors (Zhou & Chen) independently searched various databases, including CNKI, Web of Science, PubMed, and PsychInfo. The search terms used were combinations of “polic*” OR “law enforcement officer” AND “China” OR “Chinese” AND “pressure” OR “stress” OR “work overload” AND “burnout” OR “fatigue” OR " exhaustion” OR " disengagement” OR " work-induced apathy” AND " coping " OR " cognitive restructuring “. The search strategy aimed to locate instances of these keywords within the titles, abstracts, and keyword sections of research articles indexed across the databases covered. Secondly, to ensure a comprehensive search, additional relevant studies were identified by searching through Google Scholar (see Supplementary A for the details). Thirdly, the reference lists of the obtained articles were thoroughly screened to identify any other potentially relevant studies that may have been missed during the initial searches.

### Inclusion criteria

To ensure that the selected studies are directly relevant to the relationship between work pressure and occupational burnout in the context of Chinese mainland police and that the statistical measures used are appropriate for synthesizing the findings across different studies. The inclusion criteria for the literature in this meta-analysis are made as follows: (a) Studies that measure at least two variables related to work pressure, occupational burnout, or coping styles and report relevant statistical measures, such as Pearson correlation coefficients or other effect sizes (e.g., *F*, *t*, Cohen’s *d*, Hegen’s *g*, and *f* values) that can be converted into Pearson correlation coefficients. (b) studies limited to those published in English and Chinese due to translation capabilities. (c) Only studies that specifically focus on police officers working within mainland China will be considered for inclusion.

### Data coding

In this meta-analysis, each included study’s relevant data were coded following specific criteria. The following information was collected for each study: (a) Author. (b) The publication year of each study was noted to identify the timeframe of the research. (c) The total number of participants in each study’s sample was recorded. (d) The proportion of female participants in each study’s sample was calculated by dividing the number of females by the total population and multiplying by 100. (e) The specific province or region where the study was conducted and where the Chinese mainland police officers worked was noted. (f) The reported correlation coefficient and other statistical measures that could be converted into correlation coefficients between work pressure, occupational burnout, or coping styles in each study were recorded. (g) The measurment tools or scales used to measure work pressure, occupational burnout, and coping styles in each study were noted.

To handle studies that reported correlations between multiple dimensions of variables or between dimensions and the total score of the scale, a specific approach was employed. For studies reporting correlations between multiple dimensions, the Fisher’s *Z* scores were calculated for each dimension’s correlation and then averaged to obtain the final bivariate correlation. For studies reporting both dimensional correlations and total score correlations, only the total score correlation was used, and it was converted to Fisher’s *Z* score.

To ensure accuracy and consistency in data coding, two researchers (Zhou & Chen) received training in meta-analysis coding and specific research topics. Coding was conducted according to a coding standard manual developed by the research team, and the reliability of the coding was quantified using various statistical measures [26]. In cases of coding discrepancies, the researchers discussed and reached a consensus to resolve any disagreements.

### Study quality evaluation

The potential bias and study quality of the included studies were assessed using the National Institutes of Health’s Quality Assessment (NIHQA) tool for observational cohort and cross-sectional studies [27]. The NIHQA tool is a widely recognized and established tool for evaluating the quality of observational studies. Each study was evaluated based on specific criteria, and a total score was calculated to determine its overall quality. According to the criteria proposed by George et al. [28], studies that scored more than 80% of the total possible score were considered to be of good quality. Studies that scored between 60% and 79% were categorized as fair quality, while those scoring below 60% were deemed to be of lower quality. Two authors ( Chen & Qu ) conducted the quality assessment work and Cohen’s *K* is 0.86 which indicates that there is a high level of agreement between the two evaluators’ scores.

### Meta-analysis procedure

The steps of data analysis are as follows. First, a three-level meta-analysis was undertaken to account for the interdependence of effect sizes (ESs) by stratifying ES variance into three hierarchical levels. These levels were defined as follows: the first level represented the variance among individual participants, the second level captured the variance among ESs originating from the same study, and the third level encompassed the variance among studies. The implementation of the three-level meta-analyses in our study was facilitated through the utilization of the metafor package in R language software [29, 30]. and the heterogeneity was assessed using the *I*^2^ and *Q* statistics [31]. Sensitivity analysis was conducted to identify potential outliers, and publication bias was assessed using funnel plots, and Egger’s test. In case of the presence of publication bias, trim-fill analysis was used for correction if there was bias. Second, Meta-regression analysis will be utilized to analyze the potential moderating effects of age, gender, research quality, and workplace location on work stress and job burnout. Thried, the two-step method proposed by Cheung and Chan [32] was employed to conduct a meta-analytic Structural Equation Modeling (MASEM) to explore the mediating effect of coping strategies on the relationship between work pressure and police occupational burnout [33].

Two-Stage Structural Equation Modeling (TSSEM) is a meta-analytic technique used to synthesize correlation matrices from multiple studies into a unified structural equation model. It involves two key steps: Stage one: Aggregates individual study correlation matrices into a single pooled correlation matrix, using multigroup structural equation modeling. This stage carefully addresses the presence of different variables across studies and manages missing data. Stage two: Applies the structural equation model to the pooled correlation matrix using Weighted Least Squares estimation(WLS), prioritizing more precise estimates. This stage tests the hypothesized model, providing insights into the relationships between variables across the compiled research. The missing value in the matrix is handled by selection matrices [34].

TSSEM allows for a robust analysis of complex relationships within a vast body of literature, ensuring a comprehensive understanding of the studied phenomena. The meta-analysis and meta-analytic structural equation model was executed using the metafor and metaSEM function packages in R language [29, 35].

## Results

**Fig. 1:**
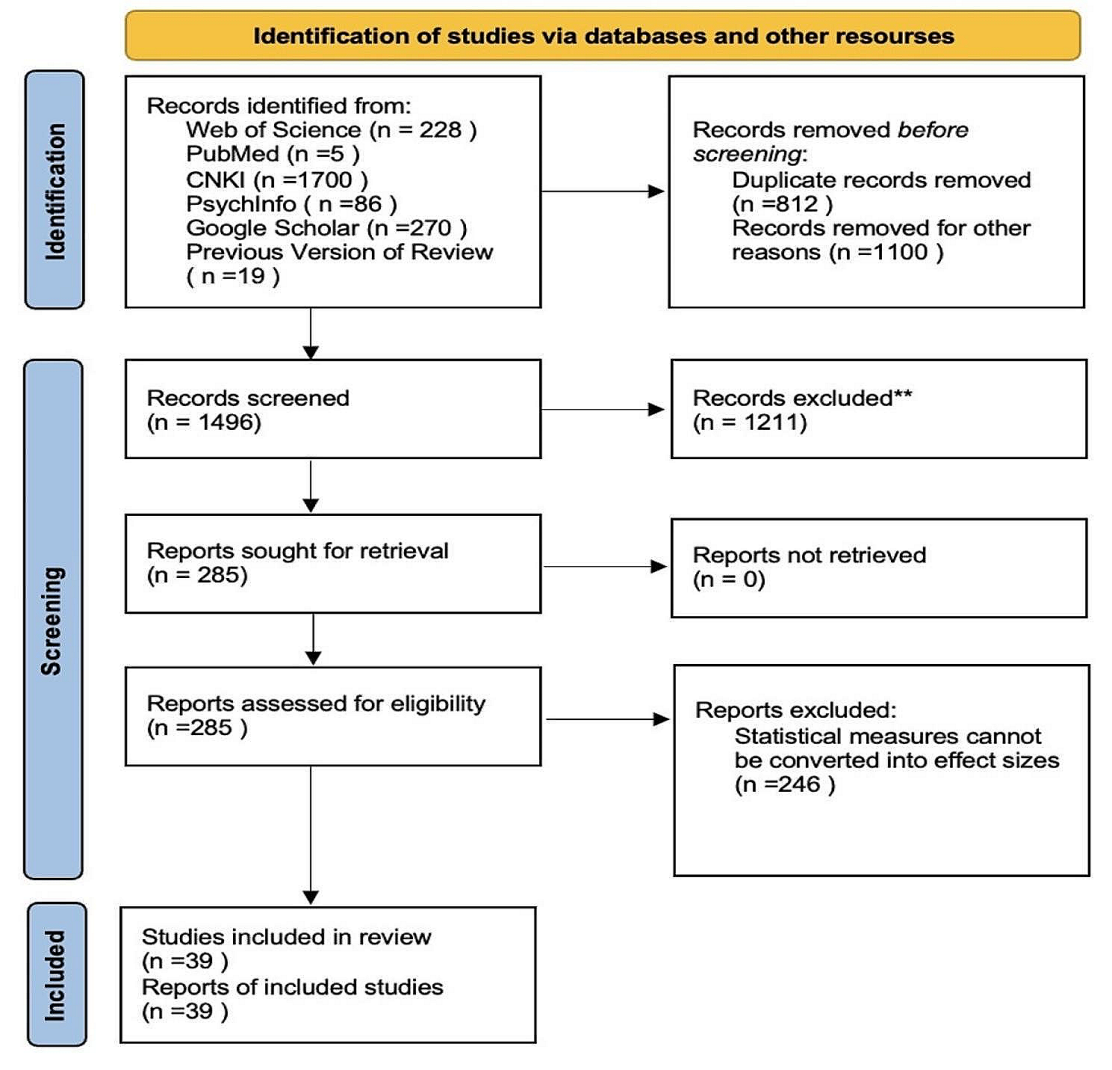
PRISMA flow chart

The initial search cut off date is March 2023. The search yielded 2308 results. After excluding 812 duplicate records and 1211 works that did not meet the inclusion criteria (e.g., non-Chinese samples, univariate studies), 285 articles were further checked based on the full-text reading. Among these, 246 studies did not provide the necessary effect size data. Finally, a total of 39 relevant studies, comprising 124 effect sizes, were included in the meta-analysis, involving a total of 14,089 police officers. The included studies were published between 2004 and 2022. The detailed process of document inclusion is illustrated in Fig. 1. The agreement on literature selection between the two coders was 89%. The reliability of coding was assessed using the Kappa statistic and the Intraclass Correlations Coefficient (ICC) magnitude, which ranged from 0.83 (Cohen’s *k*) to 0.98 (ICC) [36, 37]. Any coding discrepancies were resolved through consensus discussion (Zhou & Chen). The detailed information on the included studies is shown in Table 1.

**Table 1:** Information of studies included in meta-analyses

Author(s)	Publication year	k	*n*	Gender	Region	Police type	Pressure measure	Burnout measure	Coping measure	Age
Zhao	2019	1	187	34	NA	CO	Other	MBI-GS	NA	35.5
Li	2012	1	476	19	SC	PSP	Other	NA	Other	35
Yao	2019	1	306	0	AH	PSP	NA	MBI-GS	Other	NA
Chen et al.	2016	1	450	0	GX	CO	NA	MBI-GS	CSQ	NA
Zhang	2012	1	398	15	HLJ	CO	NA	MBI-GS	CSQ	40.5
Chen	2012	1	216	12	SC	PSP	Other	NA	Other	38.5
Wang	2017	1	882	20	SX	PSP	Other	NA	CSQ	32.12
Deng	2016	1	195	15	HN	PSP	Other	NA	Other	30
Li	2009	1	210	11	YN	PSP	Other	NA	Other	35.5
Yi	2008	1	245	16	SD	PSP	NA	MBI-GS	CSQ	30.5
Li et al.	2010	1	388	NA	XUAR	PSP	Other	MBI-GS	NA	NA
Yang	2008	1	905	12	Mixed	PSP	Other	NA	Other	34
Chen	2006	1	180	13	ZJ	PSP	NA	MBI-GS	CSQ	NA
Sun	2016	1	270	13	Tibet	PSP & SWAT	Other	MBI-GS	NA	30.5
Hou	2012	1	211	NA	HA	CO	Other	NA	CSQ	36
Chen & Ding	2014	1	247	NA	ZJ	PSP	Other	NA	Other	NA
Zhang	2011	1	571	NA	HB	PSP	Other	MBI-GS	NA	NA
Xie et al.	2010	1	180	19	ZJ	PSP	EIR-Q	NA	CSQ	35.5
Wang et al.	2014	1	331	34	Mixed	CO	EIR-Q	MBI-GS	NA	34.4
Wang et al.	2014	1	521	NA	ZJ	PSP	EIR-Q	NA	Other	NA
Huang	2020	1	340	0	JS	PSP	NA	MBI-GS	Other	NA
Gao et al.	2022	1	1024	33	LN	CO	Other	Other	NA	NA
Hang et al.	2012	3	221	20	AH	PSP	NA	MBI-GS	CSQ	36
Ma	2017	3	231	20	NA	PSP	EIR-Q	MBI-GS	Others	25
Yang et al.	2010	3	970	0	FJ	CO	Other	MBI-GS	CSQ	33.8
Liu	2009	3	98	NA	GZ	PSP	NA	MBI-GS	CSQ	40
Zhang	2009	3	249	27	JX	CO	EIR-Q	Other	NA	NA
Zheng	2013	3	239	13	Mixed	PSP	Other	NA	CSQ	32.6
Wang et al.	2007	3	378	18	SD	PSP	Other	MBI-GS	NA	34.2
Xiong	2019	3	241	NA	JX	PSP	NA	Others	NA	35.5
He	2012	4	334	66	Mixed	CO	EIR-Q	Others	NA	32.77
Zhao	2010	4	393	7	BJ	PSP	EIR-Q	Other	NA	40.5
Gong	2011	4	226	NA	NA	PSP	NA	MBI-GS	CSQ	NA
Yang et al.	2021	6	251	NA	SC	PSP	EIR-Q	MBI-GS	NA	NA
Zhang	2008	9	251	15	GD	PSP	Others	NA	CSQ	35.5
Zhang & Guo	2011	12	274	23	SX	PSP	NA	MBI-GS	CSQ	35.5
Liang	2004	12	340	20	SH	PSP	EIR-Q	Others	NA	37.83
Fang	2010	12	272	12	GD	PSP	NA	Others	CSQ	30
Pan	2014	15	388	36	SD	PSP	Other	NA	Other	NA

Note: 1. Region: XUAR = Xinjiang Uyghur Autonomous Region; SC = Sichuan; AH = Anhui; GX = Guangxi; HL = Heilongjinag; SN = Shaanxi; HN = Hunan; YN = Yunnan; SD = Shandong; HA = Henan; BJ = Beijing; ZJ = Zhejiang; XZ = Tibet; JS = Jiangsu; HB = Hebei; LN = Liaoning; FJ = Fujian; GZ = Guizhou; JX = Jiangxi; NX = Ningxia; SH = Shanghai; GD = Guangdong; Mixed = more than two provinces. 2. Gender = femal number/total samplenumber*100; 3. Police type: CO = correctional officer; PSP = public security police; PSP& SWAT = public security police & SWAT team; NA = not reported. 4. Scales: ERI-Q = Effort-Reward Imbalance Questionnaire, MBI-GS = Maslach burnout inventory-general survey; CSQ = Client satisfaction questionnaire; Others = Scales used less frequent (< 3 times)

### Results of study quality evaluation

Among the included studies, thirteen studies were classified as low quality, fifteen as medium, and eleven as high quality (see Supplementary B for the details). Incorporating studies across quality spectra requires appropriate statistical techniques and adjustments for quality weighting [38] to manage the potential impact on results. Consequently, we conducted sensitivity analyses to examine the influence of study quality on effect size.

### Main effect

The main effects analysis included 19 relevant studies and 50 effect sizes after eliminating one study due to an abnormal effect size detected through sensitivity analysis (studentized residuals > 2.5 and Cook’s d value > 0.4). We employed a random effects model for the meta-analysis, as we anticipated the presence of moderators that might contribute to the heterogeneity of effect sizes. The combined effect size after eliminating the outlier is *r* = 0.410, with a 95% confidence interval of [0.347, 0.469]. According to Lipsey and Wilson’s criterion, a correlation coefficient greater than 0.4 is considered a high correlation [39]. The results support Hypothesis 1, confirming a high positive correlation between job work pressure and police occupational burnout.

**Fig. 2:**
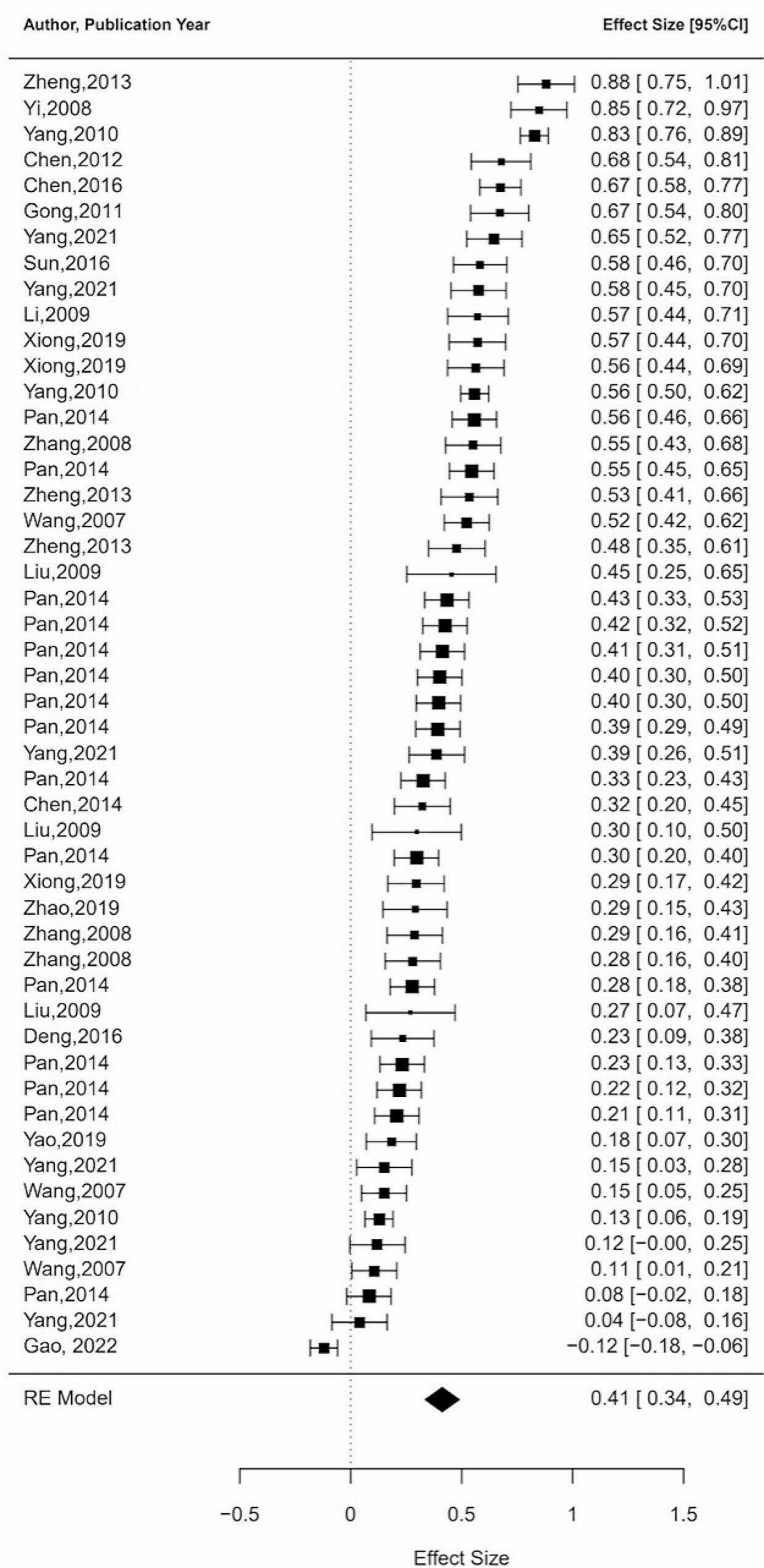
Forest plot of work pressure-occupational burnot meta-analysis

**Fig. 3:**
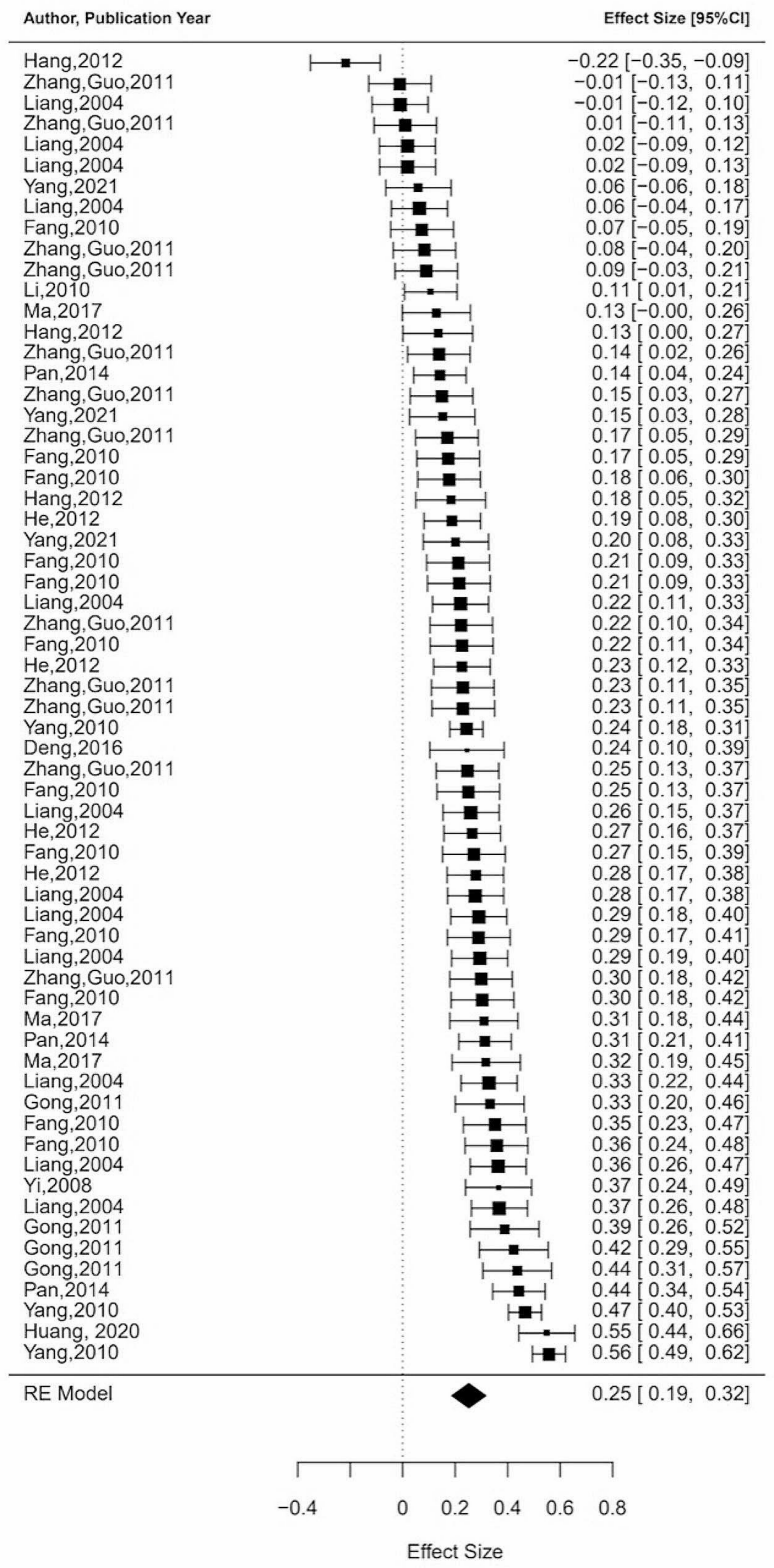
Forest plot of positive coping-occupational burnot meta-analysis

**Fig. 4:**
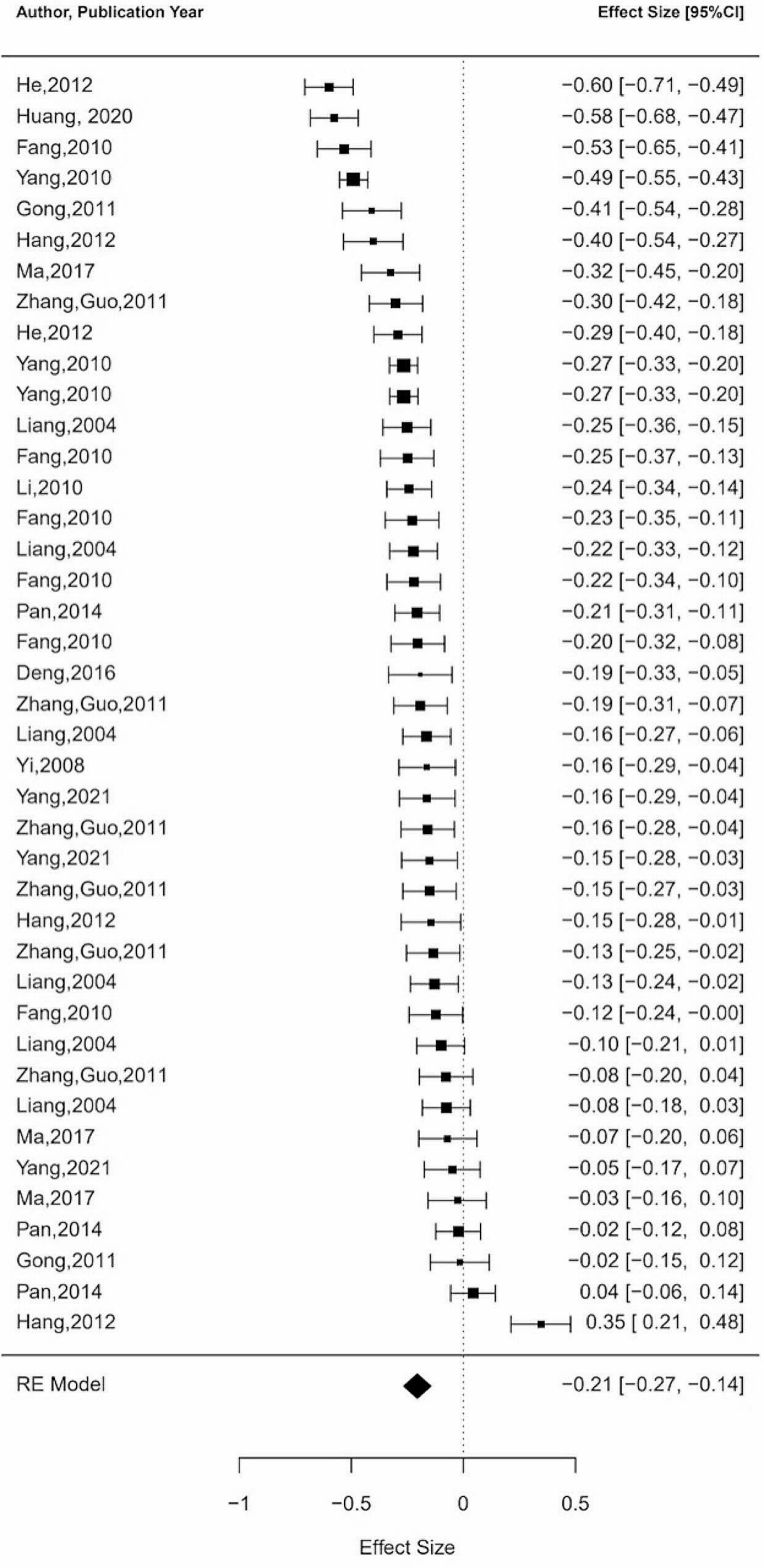
Forest plot of negative coping-occupational burnot meta-analysis

Additionally, to further support the structural equation modeling meta-analysis that follows, we also conducted a supplementary analysis to obtain the pooled correlation coefficients between positive and negative coping strategies and occupational burnout through meta-analysis. Results indicate that positive coping is significantly negatively correlated with occupational burnout, while negative coping is significantly positively correlated with occupational burnout. (see Table 2 for details). The forest plots were also conducted to illustrate the range and distribution of effect sizes of the above three meta-analyses (see Figs. 2, 3 and 4). Due to the limited number of studies included on relationship between job stress, positive coping, and negative coping, conducting a meta-analysis would result in lower statistical power, hence we did not perform meta-analyses on these aspects.

### Result of publication bias testing and heterogeneity testing

**Fig. 5:**
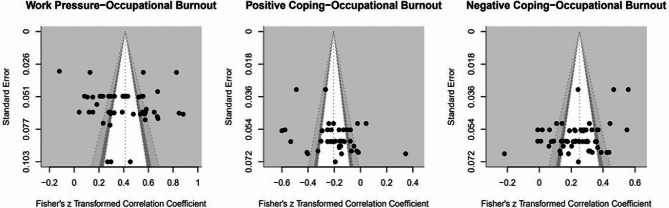
Funnel plots of three meta-analyses

To assess publication bias, three funnel plot diagrams (Fig. 5) were examined, and three Egger’s tests were conducted in all three models. The result indicated no significant publication bias. The three random effects models revealed heterogeneity among the included studies (see Table 2). This indicates that factors other than sampling error may be contributing to the observed heterogeneity in the study. Further exploration and analysis of potential sources of heterogeneity will be essential to better understand the variability across the included studies.

**Table 2:** Pooled Effect Sizes of Associations between Work Pressure, Coping Styles, and Occupational Burnout

Model	k	*n*	#ES	*r*	95% CI	${\varvec{I}}_{\mathbf{l}\mathbf{e}\mathbf{v}\mathbf{e}\mathbf{l}2}^{2}$	${\varvec{I}}_{\mathbf{l}\mathbf{e}\mathbf{v}\mathbf{e}\mathbf{l}3}^{2}$	${\varvec{\upsigma }}_{\mathbf{l}\mathbf{e}\mathbf{v}\mathbf{e}\mathbf{l}2}^{2}$	${\varvec{\upsigma }}_{\mathbf{l}\mathbf{e}\mathbf{v}\mathbf{e}\mathbf{l}3}^{2}$	Q	z
1	19	6141	50	0.410^***^	[0.347, 0.469]	79.44%	14.73%	0.040	0.07	1046.45^***^	0.41
2	13	3947	41	− 0.203^**^	[-0.260, − 0.143]	78.11%	11.69%	0.023	0.004	386.81^***^	1.70
3	13	3947	63	0.247^***^	[0.1850, 0.307]	43.89%	42.76%	0.010	0.010	449.53^***^	-0.83

Note. 1 = Work pressure - occupational burnout meta-analysis model; 2 = Positive coping - occupational burnout meta-analysis model; 3 = Negative coping- occupational burnout meta-analysis model; k = number of studies; n = number of samples; #ES = number of effect sizes; *r* = mean effect size;95%CI = 95% confidence interval of *r*; ${I}_{\text{l}\text{e}\text{v}\text{e}\text{l}2}^{2}$= percentage of variance distributed at within-study level; ${I}_{\text{l}\text{e}\text{v}\text{e}\text{l}3}^{2}$ = percentage of variance distributed at between-study level; ${{\upsigma }}_{\text{l}\text{e}\text{v}\text{e}\text{l}2}^{2}$ = variance of ESs extracted from the same study;${{\upsigma }}_{\text{l}\text{e}\text{v}\text{e}\text{l}3}^{2}$ = variance of ESs between studies; *Q* = *Q* statistical magnitude used to test the heterogeneity of effect size; *z* = Eggre’s test *z*; ^*^p < 0.05; ^**^p < 0.01; ^***^p < 0.001

### Result of meta-regression

To examine the potential moderating effects of age, gender, publication year, pressure measurement, burnout measurement, coping measurement, study quality, and the type of police force, meta-regression analyses were conducted. We performed dummy coding for all the categorical variables before meta-regression. The results showed that no variable significantly moderates the overall relationship between occupational stress and burnout (see Table 3).

**Table 3:** Results of moderators for the effect sizes

Moderator	b	SE
Age	0.039	0.028
Gender	-0.016	0.192
Publication year	-0.026	0.015
Pressure measurement (EIR-Q as reference category)		
Other	-0.016	0.008
Community sample	0.098	0.165
Burnout measurement (MBI-GS as reference category)		
Other	-0.061	0.034
Coping measurement (CSQ as reference category)		
Other	-0.081	0.032
Study quality (high quality as reference category)		
Middle quality	0.056	0.021
Low quality	0.047	0.017
The type of police force (PSP as reference category)		
CO	0.09	0.032

Note. CO = correctional officer; PSP = public security police; PSP& SWAT = public security police & SWAT team; NA = not reported. 4.Scales: ERI-Q = Effort-Reward Imbalance Questionnaire, MBI-GS = Maslach burnout inventory-general survey; CSQ = Client satisfaction questionnaire; Others = Scales used less frequent (< 3 times)

### Result of mediation effect model

Given that the samples in this study were drawn from different provinces in China, encompassing diverse police ranks and sex ratios, these factors are likely contributors to the observed heterogeneity. To account for the heterogeneity, a random effects model is employed to estimate the combined correlation matrix. The *Q* statistic (*p* < 0.01) and ${I}^{2}$ (73–88%) values indicate significant heterogeneity in the correlation matrix.

Subsequently, a structural equation model is constructed using the combined correlation matrix (Table 4), with work pressure as the independent variable, positive coping style, and negative coping style as the mediating variables, and occupational burnout as the dependent variable. The model is a saturated model, and thus the model fitting index is not presented. The regression coefficients are shown in Table 5. From the model results, it is observed that with the inclusion of coping strategies, the product of the path coefficients indicating the influence of work pressure on occupational burnout through negative coping strategies is significant (indirect effect size = 0.03, 95% CI = [0.01, 0.05], *p* < 0.05). Additionally, the direct effect on occupational burnout remains significant (β = 0.41, 95% CI = [0.30, 0.51], *p* < 0.01). Thus, it can be inferred that the partial mediation effect of negative coping strategies positively mediates the impact of work pressure on occupational burnout among Chinese police officers. However, the path coefficient indicating the influence of work pressure on occupational burnout through positive coping strategies is not significant, indicating the absence of a mediating effect. Hypothesis 2b is validated.

**Table 4:** The pooled correlation matrix

	Work pressure	Positive coping style	Negative coping style
Positive coping style	-0.17^*^ [-0.32,-0.02]		
Negative coping style	0.24^***^ [0.13,0.34]	-0.04 [-0.21,0.12]	
occupational burnout	0.45^***^ [0.35, 0.55]	-0.14^*^ [-0.27,-0.02]	0.23^***^ [0.17, 0.29]

Note: ^*^ indicates *p* < 0.05; ^**^ indicates *p* < 0.01; [ ] denotes the 95% confidence interval of the correlation coefficient

**Table 5:** The regression coefficients and indirect effect sizes of the mediation model

Path	Coefficient	95% CI
Work pressure → Positive coping style	-0.17^**^	[-0.32,-0.02]
Work pressure → Negative coping style	0.24^**^	[0.13, 0.34]
Positive coping style → Occupational burnout	-0.07	[-0.21, 0.08]
Negative coping style → Occupational burnout	0.13^*^	[0.05, 0.21]
Work pressure → occupational burnout(direct effect)	0.41^***^	[0.30, 0.51]
Work pressure → Positive coping style → Occupational burnout(indirect effect)	0.01	[-0.02, 0.04]
Work pressure → negative coping style → Occupational burnout(indirect effect)	0.03^*^	[0.01, 0.05]

Note: Path coefficients are standardized regression coefficients or products; ^*^ indicates *p* < 0.05; ^**^ indicates *p* < 0.01, ^***^ indicates *p* < 0.001; 95% CI represents the 95% confidence interval of the regression coefficient

## Discussion

The study’s findings provide valuable insights into the relationship between police job work pressure and occupational burnout. It supported the Job Demands-Resources (JD-R) Theory in the group of police officers and expanded the application of the Transactional Model of Stress and Coping in explaining occupational burnout [40, 41]. The mediating role of negative coping style revealed the underlying mechanism of the relationship between work pressure and occupational burnout. The detailed discussion is as follows:

### Police work pressure and occupational burnout

Based on an extensive meta-analysis of 39 literature sources, 68 independent sample studies, and a total of 19,980 subjects from diverse police samples across China, this study revealed a medium to high positive correlation between work pressure and occupational burnout among Chinese police officers. These findings are consistent with previous individual studies by Wang et al. and Zheng [42, 43]. Notably, the relationship between work pressure and occupational burnout was found to be relatively stable, unaffected by gender, region, and study quality, indicating a consistent pattern across different contexts.

This robust positive correlation aligns seamlessly with the core tenets of the Job Demands-Resources (JD-R) theory and the Conservation of Resources (COR) theory [44, 45]. The JD-R model posits that excessive job demands, such as heavy workloads, time constraints, and exposure to dangerous conditions, gradually deplete the psychological and emotional coping resources of police officers over time, leading to burnout. Empirical research has consistently demonstrated that the frequent demands inherent in police work, including high call volumes, mandatory overtime, and exposure to danger, contribute to emotional exhaustion and cynicism [46, 47]. COR theory further elucidates that police officers must invest substantial effort and energy to cope with these sustained demands, gradually depleting their coping reserves and resulting in stress. Studies indicate that the daily expenditure of resources required to manage work-related pressures such as danger and trauma can exhaust officers’ reserves and eventually lead to burnout [48].

In summary, the inherent high demands of modern policing deplete officers’ psychological resources and diminish their coping abilities, providing robust theoretical and empirical support for the observed association between work pressure and occupational burnout in this study. Interventions aimed at mitigating occupational burnout should prioritize the modification of excessive job demands and enhancing the coping abilities of police officers to prevent resource depletion.

A potential explanation for the consistent association between work demands and burnout across regions with varying pay levels is that police salaries have stagnated in recent years, with no significant increases even after adjusting for inflation, according to data from the Bureau of Labor Statistics [49]. This alignment may normalize the effects of absolute pay differences on burnout. Additionally, factors like perceived fairness of pay and job satisfaction may better capture the impacts of compensation on stress appraisals [47]. As this study did not assess officers’ subjective evaluations of their remuneration, these unmeasured perspectives may account for the lack of moderating effects. The stability of the work pressure-burnout link across regions implies occupational demands play a greater role in burnout than geographic pay discrepancies. However, future research should directly assess officers’ perceptions of pay equity and organizational justice regarding compensation to better understand if remuneration conditions influence the experience of work stress. Investigating multiple aspects of compensation beyond absolute pay rates can provide further insight into this issue.

This study explored whether the relationship between work pressure and burnout differs across genders, as the demands of police work may vary for male and female officers. However, the meta-analysis did not find a significant moderating effect for gender. A potential reason is the underrepresentation of women in the source studies, which comprised predominantly male samples. Prior research on mixed-gender police samples indicates female officers face unique stressors like discrimination, harassment, and work-family conflict that contribute to burnout [50]. As women only constituted a small proportion of participants across the samples synthesized, gender differences in the experience of work stress may have been obscured. Cautions should be taken in interpreting the lack of moderating effects given this limitation. Further research utilizing more gender-balanced samples could provide greater insight into whether the work pressure-burnout association substantively differs between male and female police officers. Investigating the distinct occupational demands faced by each gender and their implications for burnout remains an important avenue for future exploration.

### The mediating role of coping style

The negative correlation found between positive coping and work pressure/burnout aligns with Lazarus and Folkman’s transactional theory, which indicates adaptive coping can alleviate strain [51]. However, contrary to predictions, this study did not find a significant moderating effect of job remuneration on the relationship between work pressure and burnout. A potential explanation from COR theory is that when work pressures become severely resource-draining, positive coping methods may no longer be effective in replenishing reserves [45]. However, negative coping did mediate this relationship, suggesting maladaptive responses like avoidance amplify burnout by allowing demands to intensify and resources to progressively deplete [52]. This highlights the need to curb maladaptive coping through training in problem-focused, support-seeking techniques.

Overall, the findings provide a more robust test of the multidimensional stress-coping process by combining work conditions and coping responses. Yet, the partial mediating effects indicate additional variables and pathways likely influence the pressure-burnout relationship. Expanding beyond coping styles, future research should explore alternative mediators like self-efficacy, perceived control, and recovery experiences, guided by theories such as the JD-R model [44]. Investigating multiple mediating mechanisms can provide a more comprehensive understanding of occupational burnout development.

### Theoretical and practical implications

This study has important theoretical implications. First, it provides empirical support for the Job Demands-Resources (JD-R) theory and Conservation of Resources (COR) theory in explaining the positive association between work stressors and burnout [36, 45]. Second, it expands the application of the Transactional Model of Stress and Coping [51] to occupational settings by demonstrating the mediating effect of negative coping. Third, this research lays the foundation for investigating additional mediators like self-efficacy and moderators like social support as proposed in JD-R theory [44]. Finally, the focus on an understudied cultural context advances theoretical understanding beyond Western settings.

From a practical standpoint, the research results can guide future interventions and offer useful references for relevant authorities to better understand the objective patterns of police work. This can lead to the implementation of appropriate measures and policies to enhance police mental health and job satisfaction. To reduce work pressure at the task assignment level, it is essential to consider the individual characteristics of police officers and match them with suitable work tasks. Moreover, targeted interventions should be implemented for police officers who show signs of occupational burnout or are experiencing severe burnout. This can include various objective measures to reduce work pressure to help alleviate burnout levels.

### Research limitations and prospects

This study, leveraging a meta-analysis combined with a structural equation model, delves into the intricate relationship between job work pressure and occupational burnout within the context of Chinese police officers. It sheds light on the mediating role of negative coping styles. While the study makes significant theoretical and methodological contributions, it is important to acknowledge certain limitations in the literature and data analysis methods, which pave the way for future research directions. Firstly, despite the presence of heterogeneity among the studies, meta-regression did not identify moderating effects of gender or region. This suggests that other unpublished variables such as police culture, training, and education received might contribute to the observed heterogeneity. Future research should therefore aim to include a broader array of primary studies and expanded data to unearth potential moderators. By incorporating a wider range of original data and employing meta-regression to test additional moderators, the boundary conditions influencing the work stress-burnout relationship in policing can be more clearly defined. Secondly, the studies focusing on the interplay between work pressure, coping style, and occupational burnout are disproportionately few. Most research tends to examine the effects of work pressure and occupational burnout, or coping style and occupational burnout, separately, rather than exploring the collective impact of all three factors. This gap in data may affect the stability of conclusions drawn from the meta-analytic structural equation model. Addressing this, future studies should investigate the interactions between work pressure and coping styles more comprehensively. Thirdly, the use of a saturated structural equation model and issues with missing values have impacted the generalizability and robustness of our results. Therefore, the conclusions drawn from our research should be interpreted with caution. Last but not least, all studies included in the meta-analysis utilize cross-sectional research methods, limiting the ability to establish causal relationships between job work pressure and occupational burnout. Future investigations should broaden their scope to explore alternative mediating paths and boundary conditions of how police job work pressure influences occupational burnout. Additionally, adopting more ecological research methodologies, such as longitudinal, quasi-experimental, and experimental studies, could offer invaluable insights into the causal dynamics between these variables.

## Conclusion

The current review provides a quantitative synthesis of the relationship between work pressure and occupational burnout among Chinese police officers and delves into the underlying mechanisms of this association. The findings suggest that work pressure plays a crucial role as an antecedent variable to occupational burnout in Chinese police settings. The mechanism underlying this influence can be explained as follows: work pressure exerts an impact on negative coping styles, and subsequently, negative coping styles contribute to the development of occupational burnout.

### Electronic supplementary material

Below is the link to the electronic supplementary material.

Supplementary Material 1

Supplementary Material 2

## Data Availability

The data presented in this study are available on request from the first author.
