# Supplementary material for: Work pressure, coping styles and occupational burnout among Chinese police officers: a meta-analytic review
Source: BMC Psychol. 2024 May 16;12:275. doi: 10.1186/s40359-024-01779-6 (PMC11100108; doi:10.1186/s40359-024-01779-6)
Supplement: Supplementary file 1 — Supplementary Material 1 [file 40359_2024_1779_MOESM1_ESM.docx]

**Search Strategies for Investigating Work Stress, Coping Mechanisms, and Burnout Among Chinese Police Officers**

| **Sources** | **Search query for studies on work stress and occupational burnout among Chinese police officers** | **Search query for studies on work stress and coping strategies among Chinese police officers** | **Search query for studies on coping strategies and occupational burnout among Chinese police officers** | **Search query for studies on work stress, coping strategies, and occupational burnout among Chinese police officers** | **Total number of articles found** |
| --- | --- | --- | --- | --- | --- |
| Web of Science | TS=("polic*" OR "law enforcement officer") AND TS=("China" OR "Chinese") AND TS=("pressure" OR "stress" OR "work overload") AND TS=("burnout" OR "fatigue" OR "exhaustion" OR "disengagement" OR "work-induced apathy") | TS=("polic*" OR "law enforcement officer") AND TS=("China" OR "Chinese") AND TS=("pressure" OR "stress" OR "work overload") AND TS=("coping" OR "cognitive restructuring") | TS=("polic*" OR "law enforcement officer") AND TS=("China" OR "Chinese") AND TS=("burnout" OR "fatigue" OR "exhaustion" OR "disengagement" OR "work-induced apathy") AND TS=("coping" OR "cognitive restructuring") | TS=("polic*" OR "law enforcement officer") AND TS=("China" OR "Chinese") AND TS=("pressure" OR "stress" OR "work overload") AND TS=("burnout" OR "fatigue" OR "exhaustion" OR "disengagement" OR "work-induced apathy") AND TS=("coping" OR "cognitive restructuring") | 228 |
| PubMed | ("police" [Title/Abstract] OR "law enforcement" [All Fields]) AND ("China" [Title/Abstract] OR "Chinese" [All Fields]) AND ("stress" [Title/Abstract] OR "work stress" [Title/Abstract] OR "work overload" [Title/Abstract]) AND ("burnout" [Title/Abstract] OR "professional burnout" [Title/Abstract] OR "fatigue" [Title/Abstract] OR "exhaustion" [Title/Abstract] OR "disengagement" [Title/Abstract] OR "work-induced apathy" [Title/Abstract]) | ("police" [Title/Abstract] OR "law enforcement" [Title/Abstract] ) AND ("China" [Title/Abstract] OR "Chinese" [All Fields]) AND ("stress" [Title/Abstract] OR "work stress" [Title/Abstract] OR "work overload" [Title/Abstract] ) AND ("coping" [Title/Abstract] OR "cognitive restructuring" [Title/Abstract] ) | ("police" [Title/Abstract] OR "law enforcement officer" [Title/Abstract]) AND ("China" [Title/Abstract] OR "Chinese" [Title/Abstract]) AND ("burnout" [MeSH Terms] OR "fatigue" [MeSH Terms] OR "exhaustion" [Title/Abstract] OR "disengagement" [Title/Abstract] OR "work-induced apathy" [Title/Abstract]) AND ("coping" [MeSH Terms] OR "cognitive restructuring" [Title/Abstract]) | ("police"[Title/Abstract] OR "law enforcement officer"[Title/Abstract]) AND ("China"[Title/Abstract] OR "Chinese"[Title/Abstract]) AND ("stress"[Title/Abstract] OR "work stress"[Title/Abstract] OR "work overload"[Title/Abstract]) AND ("burnout"[Title/Abstract] OR "professional burnout"[Title/Abstract] OR "fatigue"[Title/Abstract] OR "exhaustion"[Title/Abstract] OR "disengagement"[Title/Abstract] OR "work-induced apathy"[Title/Abstract]) AND ("coping"[Title/Abstract] OR "cognitive restructuring"[Title/Abstract]) | 5 |
| PsychInfo | ("polic*" OR "law enforcement officer") AND ("China" OR "Chinese") AND ("pressure" OR "stress" OR "work overload") AND ("burnout" OR "fatigue" OR "exhaustion" OR "disengagement" OR "work-induced apathy") | ("polic*" OR "law enforcement officer") AND ("China" OR "Chinese") AND ("pressure" OR "stress" OR "work overload") AND ("coping" OR "cognitive restructuring") | ("police" OR "law enforcement officer") AND ("China" OR "Chinese") AND ("burnout" OR "fatigue" OR "exhaustion" OR "disengagement" OR "work-induced apathy") AND ("coping" OR "cognitive restructuring") | ("polic*" OR "law enforcement officer") AND ("China" OR "Chinese") AND ("pressure" OR "stress" OR "work overload") AND ("burnout" OR "fatigue" OR "exhaustion" OR "disengagement" OR "work-induced apathy") AND ("coping" OR "cognitive restructuring") | 86 |
| Google Scholar | ("police stress" OR "law enforcement stress") AND "China" AND ("occupational burnout" OR "work-related fatigue" OR "job exhaustion") AND ("stress coping strategies" OR "cognitive behavioral coping") | ("police stress" OR "law enforcement stress" AND "China") AND ("work pressure" OR "occupational stress" OR "work overload") AND ("coping strategies" OR "cognitive restructuring") | ("police stress" OR "law enforcement stress") AND ("China" OR "Chinese") AND ("occupational burnout" OR "job fatigue" OR "professional exhaustion") AND ("stress coping strategies" OR "cognitive behavioral therapy") | ("police stress" OR "law enforcement stress") AND ("China" OR "Chinese") AND ("occupational burnout" OR "job fatigue" OR "professional exhaustion") AND ("stress coping strategies" OR "cognitive behavioral therapy") AND ("coping" OR "cognitive restructuring") | 270 |
| CNKI (Chinese) | SU = ("police stress" OR "law enforcement stress") AND ("China" OR "Chinese") AND ("occupational burnout" OR "job fatigue" OR "professional burnout") | SU%=("police stress" OR "law enforcement stress") AND ("China" OR "Chinese") AND ("coping") AND ("coping" OR "cognitive restructuring") | SU%=("police stress" OR "law enforcement stress") AND ("China" OR "Chinese") AND ("occupational burnout" OR "job fatigue" OR "professional burnout") AND ("coping") AND ("coping" OR "cognitive restructuring") | SU%=("police stress" OR "law enforcement stress") AND ("China" OR "Chinese") AND ("occupational burnout" OR "job fatigue" OR "professional burnout") AND ("coping") AND ("coping" OR "cognitive restructuring") AND ("occupational burnout" OR "job fatigue" OR "professional burnout") | 1700 |
